# Incidence of Voice Disorders among Private School Teachers in Taiwan: A Nationwide Longitudinal Study

**DOI:** 10.3390/ijerph19031130

**Published:** 2022-01-20

**Authors:** Bo-Lei Chen, Ya-Yun Cheng, Cheng-Yu Lin, How-Ran Guo

**Affiliations:** 1 Department of Occupational and Environmental Medicine, National Cheng Kung University Hospital, College of Medicine, National Cheng Kung University, Tainan 704, Taiwan; larry38165@hotmail.com; 2 Department of Environmental Health, Harvard University T.H. Chan School of Public Health, Boston, MA 02115, USA; b507092063@tmu.edu.tw; 3 Department of Environmental and Occupational Health, College of Medicine, National Cheng Kung University, Tainan 704, Taiwan; 4 Department of Otolaryngology, National Cheng Kung University Hospital, College of Medicine, National Cheng Kung University, Tainan 704, Taiwan; yu621109@ms48.hinet.net

**Keywords:** voice disorders, teachers, incidence

## Abstract

*Background:* Teachers are more likely to use a loud voice at work than the general working population, but few longitudinal studies have been conducted on their risk of voice disorders. The occurrence of voice disorders in private school teachers was assessed by using the Longitudinal Health Insurance Database 2000 of Taiwan, which contains information on a random sample of 1 million beneficiaries of National Health Insurance. *Methods:* This study included private school teachers who were under 35 years old and newly employed between 2000 and 2010, and used workers with other occupations as the comparison cohort. Patients with voice disorders were identified using diagnostic codes on insurance claims. Cox proportional hazards regressions were applied to obtain relative risk estimates. *Results:* After adjusting for age, sex, income, and comorbidities of sinusitis and laryngitis, private school teachers had a higher risk of developing voice disorders (hazard ratio [HR] = 1.58; 95% confidence intervals: 1.43–1.75). In addition, the finding that elementary and high-school teachers had a higher risk than college teachers (HR: 2.56 vs. 1.44) and the pattern of increases in cumulative incidence over time supported a dose–response relationship between teaching and voice disorders. *Conclusions:* Private school teachers had higher risks of voice disorders. The results support the causality between occupation and voice disorders in teachers.

## 1. Introduction

Using a loud voice is essential for some occupations, such as teachers, instructors, singers and telemarketers. Voice disorders are often reported among these occupations [1,2,3,4,5], and teachers are more often studied than the others. A cross-sectional study in the United States on a random sample found that teachers had a higher prevalence of vocal symptoms (11.0%) than that in those who were not teachers (6.2%) and that the lifetime prevalence of voice disorders in teachers (57.7%) was also higher than that in the non-teacher reference group (28.8%) [6].

Several risk factors of voice disorders in teachers were identified in previous studies, including sex, upper airway problems, caffeine consumption, speaking loudly, and the number of classes per week [7]. The most common structural problems of voice disorders were found to be Reinke's edema and vocal nodules in a study on the basis of videolaryngoscopy [8], and vocal cysts, polyps and nodules were believed to be related to vocal abuse [9]. Occupation-related voice disorders are recognized as occupational diseases in some countries. For example, Poland recognizes structural vocal disorders as compensable occupational diseases if they fit the following criteria: (1) a definite diagnosis of hard vocal nodules, secondary hypertrophic changes in vocal folds, paresis of vocal fold adductor/tensor muscles accompanied by the phonatory insufficiency of the glottis and permanent dysphonia, (2) excessive voice workload for 15 years or more, and (3) a maximal induction period of 2 years [10].

In Taiwan, a study used the National Health Insurance Research Database (NHIRD), which covered more than 99.9% of the whole population, to assess the prevalence of voice disorders identified by the diagnostic codes on insurance claims from 2006 to 2014 and found that it was 3.6% among the adult population [11]. Voice disorders were more common in women and those between 20 and 40 years of age, but epidemiologic studies on teachers were limited [12]. Besides, few previous studies on voice disorders among teachers used a longitudinal study design, which can provide estimates of person–year incidence rates, not just prevalence, and demonstrate the risk by taking into account the increased risk associated with aging. Furthermore, most previous studies depended on questionnaires to identify cases, which were self-reported in general. Therefore, this longitudinal study with diagnosis made by physicians was conducted to provide a more solid assessment of the causality between working as a teacher and the occurrence of voice disorders.

## 2. Materials and Methods

A retrospective cohort study was conducted using data provided by the National Health Insurance of Taiwan, which covers more than 99.9% of the population. This study focused on private school teachers because teachers of public schools have additional insurance coverage. The longitudinal study design applied in this study was able to identify patients who suffered from new-onset voice disorders during the follow-up period.

### 2.1. Database

The NHIRD of Taiwan included registration files and original claim data for reimbursement by the National Health Insurance and was constructed for research applications. The Longitudinal Health Insurance Database 2000 (LHID2000) is a specific data subset which contains all the original claim data on a representative cohort of 1 million individuals randomly sampled from the 23.75 million beneficiaries in the NHIRD [13].

On each individual, LHID2000 includes an encrypted personal identification number, sex, birthday, dates of hospitalization and outpatient services, and diagnoses coded according to International Classification of Diseases, Ninth Revision, Clinical Modification (ICD-9-CM). It also includes date of insurance, insurance transfer date, insurance surrender date, occupation codes (Table S1), and monthly salary. The encrypted personal identification number can be linked to claim data, which covers the period from 1 January 2000 to 31 December 2010.

### 2.2. Participants

Two study cohorts and one comparison cohort were recruited to assess the risk of voice disorders in teachers. One study cohort consisted of teachers of private elementary and high schools (study cohort 1), and the other consisted of private college teachers (study cohort 2). Those who were between 22 and 35 years of age on 1 January 2000 were included, but those who had worked in private schools before 1 January 2000 and those who had acquired voice disorders before being a teacher were excluded.

The comparison cohort consisted of workers who had never worked in private schools and were between 22 and 35 years of age on 1 January 2000.

In all three cohorts, those who had acquired voice disorders before 1 January 2000 were excluded.

### 2.3. Measurements

Voice disorders were identified using ICD-9-CM codes 478.4 (polyp of vocal cord or larynx) and 478.5 (other diseases of vocal cords: vocal nodules, vocal cyst), and those who received hospitalization or outpatient services between 1 January 2000 and 31 December 2010 were included as cases. The date when the target diagnosis was first made was recognized as the date of diagnosis.

The time between the date of insurance and the date of diagnosis was defined as the follow-up period. For individuals without voice disorders, the follow-up period was defined as the time between date of insurance and the insurance surrender date or 31 December 2010.

The covariates collected at baseline included sex, age, income, and job tenure. Income was categorized as low (monthly income < New Taiwan Dollar [NTD] 15,840), medium (monthly income between NTD 15,840 and 25,000), and high (monthly income > NTD 25,000) according to the insurance premium, to represent the socioeconomic status [14]. For the study cohorts, job tenure was defined as the period of time working at private schools between 1 January 2000 and 31 December 2010. For the comparison cohort, job tenure was defined as the longest period of time working in a given occupation. Voice disorders related comorbidities such as gastroesophageal reflux disease (GERD), sinusitis, laryngitis, asthma, mania, major depression, and anxiety disorder were identified by ICD-9-CM codes of diagnosis (Table S2).

### 2.4. Statistical Analysis

The Kaplan-Meier method was applied to demonstrate cumulative incidence of voice disorders, and the log-rank test was applied to evaluate the differences. Cox proportional hazards regressions were used to estimate the hazard ratio [HR] of voice disorders. Variables that were associated with a *p* value less than 0.15 in the univariate analyses were included in the multivariate analyses.

SAS for Windows, Version 9.4 (SAS Institute, Cary, NC, USA) and SPSS for Windows, Version 17.0 (SPSS. Inc., Chicago, IL, USA) were used for data analyses. A two-tailed *p* value of less than 0.05 was considered statistically significant.

## 3. Results

### 3.1. Basic Characteristics and Health Conditions of the Cohort Members

**Figure 1:**
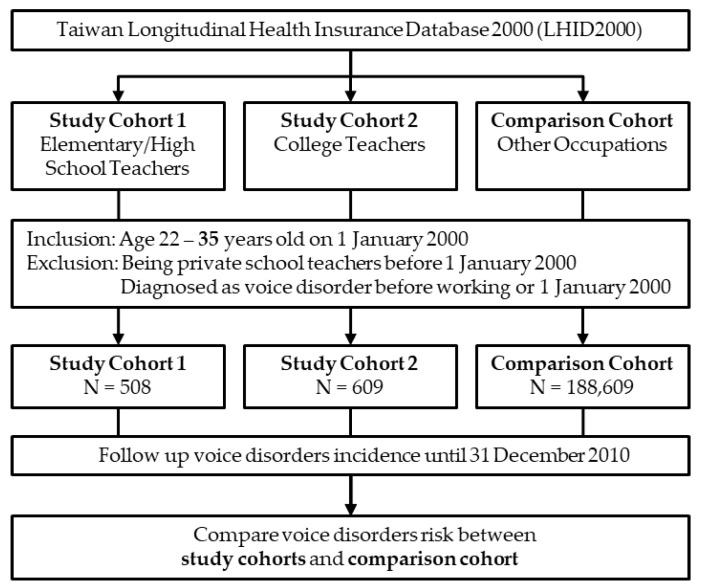
Flow chart of this study.

Through the selection process, study cohort 1 included 508 members, study cohort 2 included 609 members, and the comparison cohort included 188,145 members (Figure 1). Among the three cohorts, elementary/high-school teachers had the youngest age on average (26.6 ± 3.5 years), highest proportion of females (64.4%), and highest cumulative incidence of voice disorders (16.9%) (Table 1). They also had the highest comorbidities of sinusitis (50.0%) and laryngitis (33.9%). College teachers had the highest monthly income on average (NTD 49,868 ± 19,409).

### 3.2. Incidence Estimation and Survival Analysis

**Figure 2:**
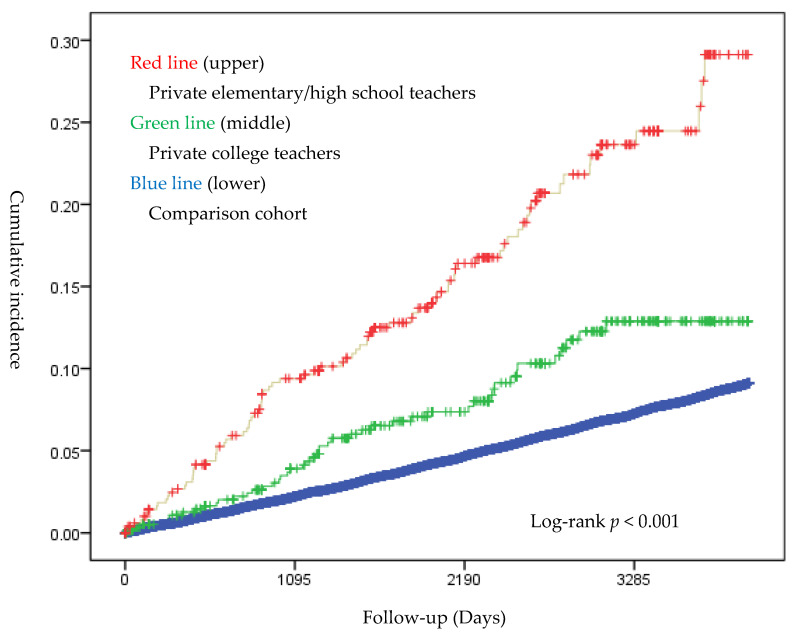
Cumulative incidence of voice disorders.

The incidence rate of voice disorders was 27.3 per 1000 person-years in elementary/high-school teachers and 13.4 per 1000 person-years in college teachers; both were higher than that in the comparison cohort (8.6 per 1000 person-years) (Table 2). Kaplan-Meier survival curves and the log-rank test showed that the occurrence of voice disorders was different among the three cohorts (Figure 2), i.e., sharper increases in the cumulative incidence with time from the comparison cohort to elementary/high-school teachers, and then to college teachers. After adjusting for age, sex, income, sinusitis, and laryngitis, the Cox proportional hazards regression showed that private school teachers had an adjusted HR of 1.58 (95% confidence interval [CI]: 1.43–1.75) for voice disorders when compared to workers with other occupations. Specifically, the adjusted HR associated with elementary/high-school teachers was 2.56 (95% CI: 2.07–3.17), and that associated with college teachers was 1.44 (95% CI: 1.08–1.92) (Table 3). When compared to college teachers, the adjusted HR associated with elementary/high-school teachers was significantly higher (1.78; 95% CI: 1.25–2.54, *p* = 0.002). The incidence was the highest in elementary/high-school teachers in the first 3 years of job tenure (8.5%).

## 4. Discussion

### 4.1. Voice Disorders in Private School Teachers

The results of this study indicated that private school teachers had a higher risk of voice disorders than workers in other occupations. A previous study found that teachers who taught below the fourth grade had a higher risk of voice disorders than those who taught above fifth grade [15]. Elementary school teachers were at a higher risk of having voice disorders than high-school teachers in another study [16]. In the current study, teachers who worked at elementary or high schools had a higher risk than those who worked at colleges. One of the reasons might be that teachers at elementary or high schools have to give more lectures than teachers at colleges. According to the regulation of teaching hours in Taiwan, teachers at elementary schools and junior high schools should give 14 to 20 h of lectures per week (Table S3). At colleges, teachers only gave 8 to 12 h of lecture per week. When working in other occupations was regarded as having nearly no teaching hours, this study supported a dose-response relationship between teaching and incidence of voice disorders, which was also supported by the sharper increases in the cumulative incidence with time from the comparison cohort to elementary/high-school teachers, and then to college, as shown in Figure 2. A dose-response relationship in turn supports a causal relationship.

From the survey of teaching quality and certification in 2001, public college teachers gave about 11 h of lecture per week, while private college teachers gave about 11.7 h of lecture per week [17]. Therefore, teachers at public colleges, although not included in this study, should also be at a high risk of developing voice disorders.

Besides teaching hours, there were other factors associated with voice disorders from previous studies. A cross-sectional study in Singapore found that the use of microphones in primary school teachers was associated with a higher risk of voice disorders [18]. A study in India found that the noise level in classrooms and stress while teaching were associated with vocal problems [19]. A study in Iran found that the number of students was also associated with voice disorders [20].

### 4.2. Other Risk Factors of Voice Disorders

In addition to occupation, the current study identified age, sex, income, laryngitis, and sinusitis as independent risk factors for voice disorders. Previous studies have shown that acute laryngitis is a common, self-limited disease generating vocal problems, and upper respiratory infection is the most common cause of acute laryngitis. In addition, upper respiratory allergies often involve the larynx, resulting in hoarseness, and they often come with symptoms of rhinitis and sinusitis [9]. Using a questionnaire survey, a study in Hong Kong also found that sinusitis and laryngitis were risk factors for voice disorders [21]. Therefore, it is reasonable that both sinusitis and laryngitis appeared to be risk factors for voice disorders.

Many previous studies also showed that women were at a higher risk of voice disorders than men [7,16,22]. There are at least several plausible reasons. First, female vocal folds have less tensile stress than those of males. Female vocal folds need more effort to keep stiffness for specific pitch. Second, the distribution of hyaluronic acid (HA) within the vocal fold extracellular matrices is different between the two sexes. HA has been found to potentially affect the damping and absorption of vocal fold collisions and thus is likely to help to protect the vocal folds. Males have a fairly stable distribution of HA throughout the depth of the vocal folds’ lamina propria, while females have less HA in the superficial layer than in the deep layer. The reduced amounts of HA in the superficial layer might predispose females to vocal fold injury and scarring, which may in turn lead to voice disorders [22].

In almost all the schools in Taiwan, teachers could obtain additional pay through teaching more hours than what were required by the regulations. On the other hand, college teachers had higher incomes than elementary/high-school teachers but gave less hours of lecture in general. Therefore, while monthly income of NTD 15,841–25,000 appeared to be a risk factor for voice disorders, monthly income over NTD 25,000 did not.

### 4.3. Voice Disorders Occurred in Early Teaching Career

The results of this study showed that more teachers were diagnosed as having voice disorders during their first 3 years on the job. About 50% of teachers got voice disorders in the first 3 years during the 11 years of study. In contrast, the other workers (comparison cohort) had similar incidence rates throughout the follow-up, which was different from the situation in teachers. A previous case-control study found that 46% of teachers with voice disorders were in their first 5 years on the job, while 28% of voice-disorder-free teachers were in their first 5 years on the job [23]. This finding was consistent with that in the current study. A possible reason is that teachers tend to use incorrect phonation methods in the beginning of their career and make corrections later on. In addition, teachers who were susceptible to voice disorders might quit their jobs earlier, and thus who stayed longer in the career tended to be heathier in terms of voice disorders. This healthy worker effect might also contribute to the observation.

### 4.4. Impacts of Voice Disorders in Teachers

Voice disorders are not fatal, but may lead to absenteeism in a substantial proportion of teachers. Teachers with voice disorders were more likely to have voice-related absenteeism from in Malaysia [24]. According to the results of a previous study [25], 29.3% of teachers with voice disorders took 1 week absenteeism, and 15.7% of teachers with voice disorders took more than 2 weeks or repeatedly 1 week absenteeism. Data from the Ministry of Education of Taiwan showed that there were 197,454 teachers at elementary and high schools and 41,949 at colleges. When the incidence rates observed in this study were applied to the statistics, it could be estimated that 1579 elementary/high-school teachers needed 1 week of rest due to voice disorders and another 846 teachers required more than 2 weeks or repeatedly 1 week of absenteeism.

### 4.5. Strengths and Limitations

This study has some limitations. First, the outcome was determined based on ICD-9-CM codes, which were recorded by physicians without confirmation, such as videolaryngoscopy or biopsy. However, it was not feasible to perform those confirmation procedures on every patient because the examinations are invasive. Alternatively, having the same ICD-9-CM code on more than one outpatient service claim during the study period could be used as the indicator of confirmed diagnosis. As a result, the proportion of patients with more than one visit was similar across the three cohorts: 46% in elementary/high-school teachers, 44% in college teachers, and 45% in the comparison cohort. Therefore, the potential misclassification would not introduce remarkable effects on the relative risk estimates observed in this study.

Second, the occupation codes included both teachers and staff, which were indistinguishable from the database. According to statistics published by the Ministry of Education of Taiwan, teachers constituted about 81% of the whole staff at private elementary/high schools and 76% at private colleges (Table S4). Besides, many private school staff members in Taiwan work as part-time or full-time teachers without a formal teaching position. Furthermore, this misclassification would lead to underestimation of the risk in teachers when they were compared to the other workers. Therefore, the potential bias would not affect the conclusion of a higher risk in teachers.

Third, data on some other risk factors were not available from the database, such as smoking and lifestyle. In particular, smoking is known as a risk factor for voice disorders. Nonetheless, according to the smoking behavior questionnaire survey of college teachers and staff conducted by the Health Promotion Administration, Ministry of Health and Welfare of Taiwan, only 1.5% of female teachers and staff were smokers. Therefore, sex could serve as an appropriate surrogate for the adjustment of smoking in this study.

This study also had several strengths. First of all, this is a large population-based cohort study. The size of the cohort allowed us to evaluate effects of some conditions that were relatively rare (such as some of the comorbidities) and adjust for the effects of many potential confounders at the same time. In addition, the study population was a random sample of the beneficiaries of the National Health Insurance of Taiwan, which covers more than 99,9% of the residents. Therefore, the current study had a representative sample of the general population, which could minimize the effects of selection bias. Furthermore, the main outcomes were diagnosed by physicians, which insured a high level of accuracy in comparison with self-reported symptoms.

## 5. Conclusions

This study observed higher risks of voice disorders in private school teachers in Taiwan, which is very likely to be also true for teachers in the public schools and teachers in other countries. The longitudinal study design and dose-response relationship between teaching and voice disorders support a causal relationship. Teachers should take cautions in the use of voice, especially at the beginning of their teaching career. Further studies should be conducted to assess the proportions of teachers who have functional voice disorders and who change their occupation consequently, as well as the course of this process.

## Figures and Tables

**Table 1:** Demographic characteristics and comorbidities of cohort members.

Characteristics	Number (%) or Mean ± SD			*p* Value
	Other Occupations n = 188,145	College Teachers n = 609	Elementary/High-School Teachers n = 508	
Sex				
Male	96,944 (51.5)	283 (46.5)	181 (35.6)	<0.001
Female	91,201 (48.5)	326 (53.5)	327 (64.4)	
Age (year)	28.0 ± 4.0	27.7 ± 4.0	26.6 ± 3.5	<0.001
Income (NTD ^1^/month)	18,041 ± 11,676	49,868 ± 19,409	36,468 ± 11,388	<0.001
≤15,840	78,847 (41.9)	1 (0.2)	1 (0.2)	<0.001
15,841~25,000	75,518 (40.1)	61 (10.0)	95 (18.7)	
≥25,001	33,895 (18.0)	547 (90.0)	412 (81.1)	
Voice disorders	15,016 (8.0)	47 (7.7)	86 (16.9)	<0.001
Comorbidity				
GERD ^2^	5048 (2.7)	19 (3.1)	15 (3.0)	0.745
Sinusitis	61,091 (32.5)	232 (38.1)	254 (50.0)	<0.001
Laryngitis	42,806 (22.7)	541 (24.8)	617 (33.9)	<0.001
Asthma	6131 (3.3)	22 (3.6)	22 (4.3)	0.351
Mania	126 (0.1)	0 (0.0)	1 (0.0)	0.688
Major depression	1643 (0.9)	7 (1.2)	5 (1.0)	0.738
Anxiety disorder	1716 (0.9)	2 (0.3)	2 (0.4)	0.150

^1^ New Taiwan Dollar; ^2^ gastroesophageal reflux disease.

**Table 2:** Comparison of risks of voice disorders.

Characteristics	Number (%) or Mean ± SD			*p* Value
	Other Occupations n = 188,145	College Teachers n = 609	Elementary/High-School Teachers n = 508	
Sex				
Male	96,944 (51.5)	283 (46.5)	181 (35.6)	<0.001
Female	91,201 (48.5)	326 (53.5)	327 (64.4)	
Age (year)	28.0 ± 4.0	27.7 ± 4.0	26.6 ± 3.5	<0.001
Voice disorders				<0.001
Occur in 3 years	3842 (2.0)	20 (3.3)	43 (8.5)	
Occur in 4~6 years	3951 (2.1)	14 (2.3)	24 (4.7)	
Occur in 7~9 years	3932 (2.1)	13 (2.1)	15 (3.0)	
Person-years	1,738,747	3515	3,146	
Incidence rate ^1^	8.6	13.4	27.3	

^1^ per 1000 person-years.

**Table 3:** Adjusted hazard ratios obtained from a Cox proportional hazards model that includes age, sex, income, sinusitis, and laryngitis.

Characteristics	Adjusted Hazard Ratio	95% Confidence Interval	*p* Value
Occupations			
Other occupations	Reference		
College teachers	1.44	1.08–1.92	0.013
Elementary/high-school teachers	2.56	2.07–3.17	<0.001
Age (continuous variable)	1.01	1.01–1.02	<0.001
Sex			
Male	Reference		
Female	2.22	2.14–2.30	<0.001
Income (NTD/month)			
≤15,840	Reference		
15,841–25,000	1.1	1.05–1.15	<0.001
≥25,001	0.99	0.95–1.02	0.450
Comorbidity			
Sinusitis	2.47	2.38–2.56	<0.001
Laryngitis	1.73	1.67–1.79	<0.001

## Data Availability

Interested researchers could obtain the NHIRD through formal application to the Health and Welfare Data Science Center, Department of Statistics, Ministry of Health and Welfare, Taiwan (http://dep.mohw.gov.tw/DOS/np-2497-113.html; accessed on 11 January 2022).

## Supplementary Materials

The following are available online at https://www.mdpi.com/article/10.3390/ijerph19031130/s1, Table S1: Occupation Codes of Longitudinal Health Insurance Database 2000. Table S2: Outcome meas-ured by International Classification of Diseases, Ninth Revision, Clinical Modification (ICD-9-CM) in the current study. Table S3: Regulations of teaching hours in Taiwan. Table S4: Numbers and percentage of private school teachers and staff in 2000.
