# Supplementary material for: Incidence of Voice Disorders among Private School Teachers in Taiwan: A Nationwide Longitudinal Study
Source: Int J Environ Res Public Health. 2022 Jan 20;19(3):1130. doi: 10.3390/ijerph19031130 (PMC8834254; doi:10.3390/ijerph19031130)
Supplement: Supplementary file 1 [file ijerph-19-01130-s001.zip › ijerph-1511393-supplementary.pdf]

Table S1. Occupation Codes of Longitudinal Health Insurance Database 2000.

Codes	Job description of The Beneficiary
A	Central government civil servant
	Public college teacher or staff
B	Local government civil servant
	Public school (except college) teacher or staff
C	Other public servant
D	Private college teacher or staff
E	Private elementary/high school teacher or staff
F	Government-owned company staff (under public insurance coverage)
G	Government-owned company staff (under labor insurance coverage)
H	Private company worker
I	Central government worker
J	Local government worker
K	Private school worker
M	Nongovernment organization worker
N	Apprentice in the vocational skill training program
O	Self-employed worker
P	Professional and skilled person, self-employed
Q	Farmer
	Fisherman

Table S2. Outcome measured by *International Classification of Diseases*, Ninth Revision, Clinical Modification (ICD-9-CM) in the current study.

ICD-9-CM	Diagnosis
**478.4**	Polyp of vocal cord or larynx
**478.5**	Other diseases of vocal cords: Abscess of vocal cords, Cellulitis of vocal cords, Granuloma of vocal cords, Leukoplakia of vocal cords, Chorditis (fibrinous) (nodosa) (tuberosa), Singers' nodes
**784.4**	Voice disturbance
**461**	Acute sinusitis
**473**	Chronic sinusitis
**464**	Acute laryngitis and tracheitis
**476**	Chronic laryngitis
**493**	Asthma
**530.11**	Reflux esophagitis
**530.81**	Gastroesophageal reflux disease
**305.0**	Alcohol abuse
**305.1**	Tobacco use disorder
**296.1**	Manic disorder, recurrent episode
**296.3**	Major depressive disorder, recurrent episode
**300.02**	Generalized anxiety disorder

Table S3. Regulations of teaching hours in Taiwan.

Level of School	Teaching Hours Per Week
Elementary school & Junior high school	Not tutors: 16–20, should not exceed 20.<br>Tutors: 10–16
Senior high school	Not tutors: 14–18<br>Tutors: 10–14
College	Vocational school: 10–12

University: 8–10

Table S4. Numbers and percentage of private school teachers and staff in 2000.

**School year: 1999**	**Teachers**	**Staff**	**Total**
Primary school	850	143	
Junior high school	318	87	
Senior high school	10,837	2316	
Vocational school	8653	2196	
**High or elementary school**	20,658 (81%)	4742 (19%)	25,400 (100%)
Junior college	6700	1385	
College	9300	2405	
University	8994	4068	
**College school**	24,994 (76%)	7858 (24%)	32,852 (100%)
